# Wet–Dry Cycles and Microstructural Characteristics of Expansive Subgrade Treated with Sustainable Cementitious Waste Materials

**DOI:** 10.3390/ma16083124

**Published:** 2023-04-15

**Authors:** Samuel J. Abbey, Samuel Y. O. Amakye, Eyo U. Eyo, Colin A. Booth, Jeremiah J. Jeremiah

**Affiliations:** 1 School of Engineering, College of Arts, Technology and Environment, University of the West of England, Bristol BS16 1QY, UK; samuel_amakye@outlook.com (S.Y.O.A.); eyo.eyo@uwe.ac.uk (E.U.E.); jeremiah.jeremiah@uwe.ac.uk (J.J.J.); 2 Centre for Architecture and Built Environment Research (CABER), College of Arts, Technology and Environment, University of the West of England, Bristol BS16 1QY, UK; colin.booth@uwe.ac.uk

**Keywords:** durability, wetting–drying, microstructural, California bearing ratio, expansive subgrade, brick dust waste, sustainable soil stabilisation

## Abstract

This work presents an experimental study on the physico-mechanical and microstructural characteristics of stabilised soils and the effect of wetting and drying cycles on their durability as road subgrade materials. The durability of expansive road subgrade with a high plasticity index treated with different ratios of ground granulated blast furnace slag (GGBS) and brick dust waste (BDW) was investigated. Treated and cured samples of the expansive subgrade were subjected to wetting–drying cycles, California bearing ratio (CBR) tests, and microstructural analysis. The results show a gradual reduction in the California bearing ratio (CBR), mass, and the resilient modulus of samples for all subgrade types as the number of cycles increases. The treated subgrades containing 23.5% GGBS recorded the highest CBR value of 230% under dry conditions while the lowest CBR value of 15% (wetting cycle) was recorded for the subgrade treated with 11.75% GGBS and 11.75% BDW at the end of the wetting–drying cycles, both of which find useful application in road pavement construction as calcium silicate hydrate (CSH) gel was formed in all stabilised subgrade materials. However, the increase in alumina and silica content upon the inclusion of BDW initiated the formation of more cementitious products due to the increased availability of Si and Al species as indicated by EDX analysis. This study concluded that subgrade materials treated with a combination of GGBS and BDW are durable, sustainable and suitable for use in road construction.

## 1. Introduction

Road subgrade materials are subjected to various weather conditions such as rain and extreme heat during spring and summer, especially in tropical areas. Subgrade materials found in shallow depths are vulnerable to continuous wetting and drying cycles in these regions [1]. The durability of the mechanical properties and the deformation behaviour of subgrade materials under these changing environmental conditions have been a concern and have been investigated using the wetting and drying cycle test [2,3]. These investigations have shown that subgrade strength decreases with an increase in wetting and drying cycles according to [4], as a result of the breakdown of interlocking bonds underpinned by constant swelling and shrinking from wetting and drying, respectively [5,6,7]. This further shows that various thresholds exist in terms of “cycles to failure” for different subgrade materials treated with different binders. The wetting and drying cycles can significantly affect the hydro-mechanical behaviour of unsaturated soils [4,8,9].

Expansive subgrades exhibit a significant increase in irreversible bulk volume upon wetting [2]. These subgrades are weak when wet and exhibit high shrink–swell behaviours, with changes in moisture content leading to movement within the subgrade capable of inducing large differential volume changes leading to cracking and other associated defects in road pavements [10,11,12,13]. Subgrades with these characteristics would fail quickly during wetting–drying cycles and hence are not suitable for use in road construction [14].

Consequently, the strength and durability of expansive subgrade materials are usually improved before construction, using chemical stabilisation processes entailing the addition of binders to the soil to improve their mechanical properties such as the California bearing ratio (CBR), the unconfined compressive strength (UCS), and the resilient modulus, and to reduce their water adsorption and, eventually, swell potential [12]. Studies have used different types of binders including cement, lime and other wastes and industrial by-products such as Silica fume, geo-textiles, brick and plastic wastes to improve on the performance of such problematic soils [15,16,17,18,19,20,21,22,23,24,25,26,27,28]. The application of these alumina and silica-rich by-product materials are well known for their strength-giving pozzolanic reaction in the presence of an activator, traditionally calcium-based. This reaction results in the formation of cementitious products, namely calcium silicate hydrate (C-H-S) gels which harden under proper curing conditions and periods to glue soil particles and fill-up pore spaces. The reduced pores and overall increase in density contribute to improving the strength and reducing the moisture absorption of the treated materials [13,29,30].

However, the durability of such stabilised material continues to be of immense concern with the current extremes in terms of weather. Researchers have continuously focused on sustainability based on durability. Several studies have moved beyond the success of strength enhancement to consider durability of such enhancement and resilience in the face of harsh climate changes. A study by Wassermann [31] on the effects of wetting–drying cycles on the performance of cement treated soils reported a higher mass loss in samples with lower binder contents with increasing cycles. Treated samples are reported to continuously get eroded with each wet–dry cycle. Durability studies on untreated and lime-treated soils showed that the higher void ratio and the increased pore size distribution were more severe in the untreated soil samples compared to lime-treated counterparts after subjecting both samples to wetting–drying cycles [32]. The increase in pore size distribution modified the compression curve of the untreated samples from a convex shape into a linear profile, indicating larger deformation under similar loading conditions after several cycles, due to a loss in interparticle bond with increasing cycles as expected [32].

In this study, the engineering properties of expansive subgrade materials were improved using sustainable waste materials by enhancing their durability to withstand large cycles of wetting–drying. Microstructural analysis via scanning electron microscopy (SEM) and mineralogical analysis via energy dispersive X-ray (EDX) were conducted on the treated subgrade materials to identify the changes in the morphology, elemental composition, and other formations in the stabilised subgrade materials. Artificially synthesised subgrade materials from a mixture of bentonite and kaolinite at various proportions were constituted to form high-plasticity-index subgrades. The subgrades were then treated using waste materials such as ground granulated blast furnace slag (GGBS) and brick dust waste (BDW) as partial replacement for cement and lime in the mix. After the subgrade materials were treated, a wetting–drying cycle test was conducted in accordance with [33,34] by recording their CBR values after each wetting and drying cycle. The result of this study provides insight into the viability of BDW in combination with GGBS as an eco-friendly binder. This is a vital step in the light of current efforts in transitioning from cement and lime-based binders to more sustainable materials to cut-down on the current carbon footprint and expand the knowledgebase in terms of alternative admixtures for sustainable infrastructural development.

## 2. Materials and Methods

### 2.1. Materials

The materials used in this study include ordinary Portland cement, lime, GGBS, BDW, bentonite, and kaolinite mixed at varying proportions to form two subgrade materials named subgrade 1 (25% bentonite + 75% kaolinite) and subgrade 2 (75% bentonite + 25% kaolinite). The choice of the composition of the subgrades was to simulate a lower and upper band in terms of expansivity of subgrades. Whereas subgrade 2 containing 75% bentonite is an extremely highly expansive soil with liquid limit of 294.07%, plastic limit of 45.38%, and a plasticity index of 248.69%. Subgrade 1 with 25% bentonite qualifies as a high plasticity soil with liquid limit of 131.26%, plastic limit of 28.24%, and a plasticity index of 102.52%. The bentonite and kaolinite used were supplied by Potclays Ltd. (Stoke-on-Trent, UK), while the cement (CEM I) used was supplied by Dragon Alfa Cement Ltd. (Bristol, UK), and in compliance with [35]. The lime used was supplied by Singleton Birch Ltd. (Barnetby, UK) and was in compliance with [36] to be classified as hydrated lime, while the BDW was supplied by Celtic Sustainable Ltd. (Ceredigion, UK) and in compliance with [37]. Furthermore, the GGBS used was supplied by Francis Flower in compliance with [38]. The sustainable waste materials used consisted of GGBS and BDW used at proportions of 23.5% GGBS and 0% BDW, and 11.75% GGBS and 11.75% BDW for both subgrade types. Compaction and Atterberg limit tests were conducted in compliance with [39,40,41] to determine the Optimum Moisture Content (OMC) and Maximum Dry Density (MDD) of untreated subgrade materials. The procedure and equipment used to conduct microstructural analysis in this study are as reported in the authors’ previous study [12,42]. The study also conducted wetting–drying cycle on the subgrade materials formulated in this study in accordance with the wetting and drying procedure described in [33]. After each cycle of wetting–drying, the CBR samples were tested in accordance with [34]. The oxide compositions of the materials are presented in Table 1.

### 2.2. Sample Preparation and Experimental Design

Wetting–drying cycle test was conducted in this study following the wetting and drying procedure outlined in [33]. A total of 21 cycles of wetting and drying were performed. Wetting–drying cycles conducted in this study were to simulate the effect of repeated wetting and drying cycles on treated road subgrade materials during the wet (flooding) and dry (drought) seasons. CBR samples were prepared in a 152 × 178 mm steel mould from 3 layers of stabilised soil each receiving 62 blows of a 2.5 kg rammer, using an automatic mechanical compaction machine. The samples were compacted at 1.2 of OMC to allow for adequate moisture for hydration and pozzolanic reaction during curing and stored at a temperature of 22 ± 2 °C in compliance with [34]. The stabilised samples were wrapped in clingfilm and cured for 28 days before subjecting to wetting–drying cycle test. After curing the samples, the clingfilm was removed and the samples were wrapped using duct tape leaving the top and bottom of the sample. This was done to hold the sample together in one piece during the wetting–drying cycles test. All CBR samples were submerged in water for 5 h at a room temperature of 20 ± 2 °C to simulate flooding during heavy rainfall. After 5 h of soaking, the samples were removed and weighed, and their mass was recorded to monitor the mass change in the sample in the course of the cycles. After the soaking period, one sample was set aside and tested for CBR (wetting cycle No 1) and the CBR value was recorded. The remaining soaked samples were then placed in an oven at a temperature of 71 ± 3 °C for a period of 42 h to simulate extreme dryness (drought) or high temperature. After 42 h the dry samples were removed, and their masses recorded. Thereafter, one sample was set aside and tested for CBR (drying cycle No 1) and the CBR value was recorded. This procedure constitutes one cycle (48 h) of wetting and drying [33,34]. The process was repeated until all 10 cycles were achieved to see the effect of wetting–drying cycles on the durability of the treated subgrades. The purpose of conducting wetting–drying test in this study was to establish a limiting number of cycles at which the CBR of the subgrade materials fall below allowable limits and hence the subgrades become unsuitable for use in road construction. A high-quality subbase material will typically have a CBR value between 80% and 100% and a subgrade with a CBR value < 2% is unacceptable for use in road construction and would require modification or treatment [12].

## 3. Results and Discussion

### 3.1. Wetting and Drying Cycles

**Figure 1:**
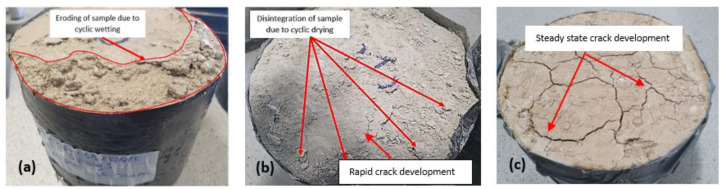
(**a**) Eroded subgrade sample due to cyclic wetting (**b**) Rapid crack development stage with loose particle following breakdown of interparticle bonds due to high temperature (**c**) Oven dry sample exhibiting steady-state crack development similar to a typical natural dry expansive subgrade.

From the results of wetting–drying cycle on subgrade samples, deeper cracks were observed for subgrade 2 (extremely high plasticity index), which was composed of 75% of bentonite compared to subgrade 1 (25% bentonite). This observation was due to the high shrinkage potential because of the high amount of bentonite content in the mix as shown in Figure 1. Weight loss in samples was observed for all mix designs as the number of cycles increased [44]. A significant loss in mass of the samples was observed from cycles 4 to 10 for both wet and dry testing conditions. This weight loss was a result of the weakening of the interparticle bonds due to the constant swelling and shrinking caused by cyclic wetting and drying. Particles of treated soil were constantly disintegrating and falling-off as the wetting–drying process progressed. This behaviour is expected and has been observed in binders used in subgrade stabilisation when exposed to wetting and drying [45]. The study by [46], showed adverse effect of increasing temperatures on stabilised soil with increasing mass loss of soil. It was observed that increased heating conditions led to a decrease in sample sizes. A drastic drop in sample mass after oven drying was observed. However, samples regained some amount of mass during the wetting processes (soaking) due to water filling the pore spaces in the samples. The occurrence of mass loss was due to the evaporation of moisture out of the sample during oven drying at 71 ± 3 °C leading to the development of microcrack which expanded following water ingress during soaking with associated swelling. The loss in bond strength and expansion of the samples during the wetting phase further expanded the cracks and increased the void ratio of the samples thereby reducing the mass of the sample as shown in Figure 1.

**Figure 2:**
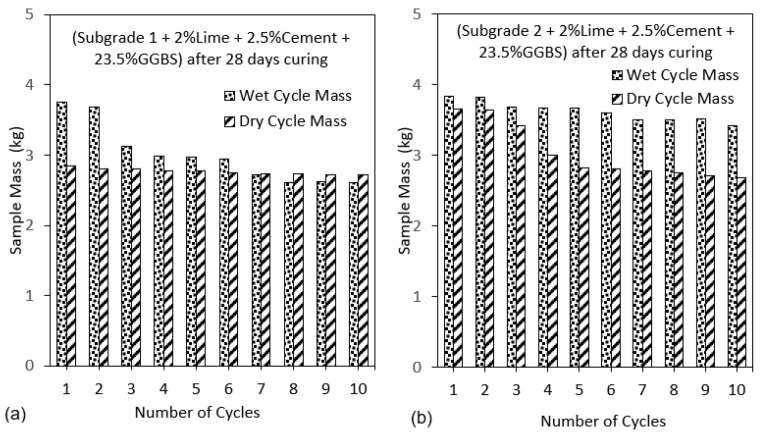
(**a**) Sample mass for subgrade 1 composed of GGBS after 28 days of curing (**b**) Sample mass for subgrade 2 composed of GGBS after 28 days of curing.

**Figure 3:**
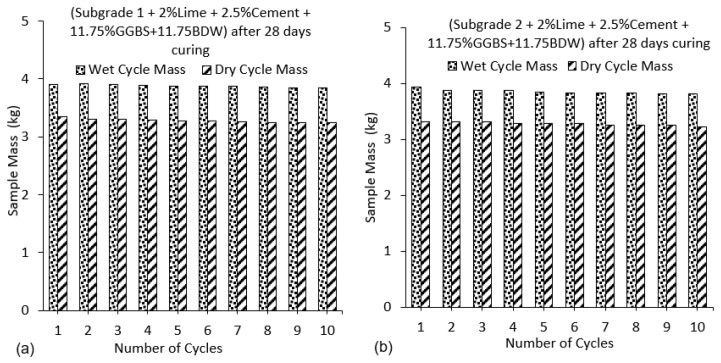
(**a**) Sample mass for subgrade 1 composed of GGBS and BDW after 28 days of curing (**b**) Sample mass for subgrade 2 composed of GGBS and BDW after 28 days of curing.

Cracking after different numbers of wetting–drying cycles was obtained in the subgrade [47]. According to the British lime association [48], more moisture loss was observed through evaporation due to heat generated during the hydration and pozzolanic reactions synonymous with soil stabilisation using cement, lime and GGBS. Two stages of crack development at the end of the drying process of the high-plasticity-index subgrade were observed-the stage of rapid crack growth and the stage of steady crack development, these stages affect the strength of the soil while reducing the soil mass [47]. This behaviour is very common in naturally existing high-plasticity-index subgrade materials when subjected to high temperatures, especially in tropical areas. When oven-dry samples were submerged in water during the wetting cycle process, water, with a higher density, quickly occupied the pores, expelling the air from within the sample hence increasing the mass of the sample as shown in Figure 1a–c. Figure 2 and Figure 3 show the gradual reduction in sample mass for subgrade materials as the number of cycles increases.

The effect of the addition of 11.75% of BDW to the binder mix can be seen from Figure 3a,b. The increase in mass loss from the use of 2% lime, 2.5%cement, and 23.5% GGBS with increasing cycles was significantly reduced from almost 10% to less than 2% for the wet cycles when 11.75% BDW was introduced into the binder. The gradual loss in mass of the samples resulted in lower moisture absorption. This was because a reduction in the mass of the stabilised soil from disintegration, led to an overall reduction in the void ratio as the wet–dry cycles progressed. The overall reduction in the void ratio also resulted in lower mass of the samples during the wetting cycle as the mass of the absorbed water was lowered in the process. The reduction in mass loss indicates further increase in the strength of the stabilised expansive soils from the addition of BDW. BDW is known for its high pozzolanic properties and have been reported to increase the strength of stabilised soils [49]. In addition, BDW known for its high specific area is very reactive as a highly pozzolanic material. The finer particle size of the BDW filled the interparticle pores, which resulted in a denser and more compact soil-binder matrix with enhanced strength and durability.

In the current study, the effect of 11.75% BDW is seen in enhancing the durability of the treated soils. From Figure 3a it seen that at the 10th cycle, the sample mass is relatively stable compared to the samples stabilised with 23.5% GGBS. This might indicate increased durability when BDW is utilised and suggests that BDW might be a useful additive in increasing the durability of stabilised samples.

### 3.2. The California Bearing Ratio (CBR)

**Figure 4:**
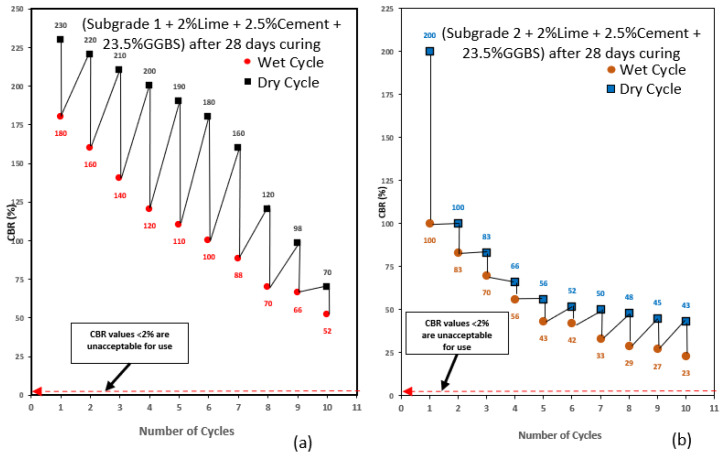
(**a**) Wetting–drying cycle results for subgrade 1 composed of GGBS after 28 days of curing. (**b**) Wetting–drying cycle results for subgrade 2 composed of GGBS after 28 days of curing.

**Figure 5:**
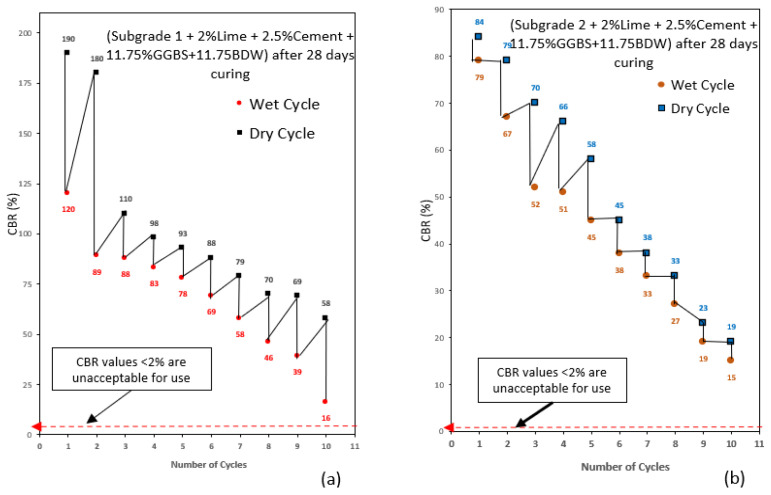
(**a**) Wetting–drying cycle results for subgrade 1 composed of GGBS and BDW after 28 days of curing. (**b**) Wetting–drying cycle results for subgrade 2 composed of GGBS and BDW after 28 days of curing.

The CBR values used in this study was the average CBR value of two samples to ensure validity and accuracy of the results. After conducting wetting–drying cycle on the treated subgrade samples, it was observed that the CBR of the subgrade samples was decreasing with an increase in the number of cycles as expected. This observation confirms the findings by [1], that subjecting soil to wetting–drying cycles caused the decrease in soil strength. The CBR value of subgrade 2 was less compared to subgrade 1. The reduction in CBR values was observed for all subgrade samples with an increase in wetting–drying cycles. The reduction in CBR value as wetting–drying increased could be attributed to the repeated swelling and shrinking due to wetting–drying resulting in loss of binder strength within the samples [1]. Very high CBR values more than twice that of wet samples were recorded for dry cycle samples. The extremely high CBR observed in dry cycles was due to the ability of high-plasticity-index clays to harden during drying under elevated temperatures. The hardening of the clay increased the CBR of the soil hence, the high CBR values were recorded for drying-cycle samples. The low CBR values recorded for wet-cycle samples compared with dry-cycle samples could be because high plasticity clay is weak in the presence of water [50]. Overall, the CBR recorded for subgrade 2 were lower compared with subgrade 1 as shown in Figure 4 and Figure 5.

This was due to the high amount of bentonite content in the mix, making it expansive and weak in compression. According to [12], subgrade materials with high bentonite content have low CBR values. Subgrade mix design composed of high GGBS content (23.5%) recorded the highest CBR values for wetting–drying cycles, making it the best performing mix in this study. The high strength values achieved with the addition of high amounts of GGBS in the mix supports some of the results presented by [12], which is because the relatively high calcium content in GGBS, the main reaction product of CSH gel is responsible for the increase in strength in a mix [51]. Studies have shown that the higher the amount of GGBS blends, the greater the strength and durability of the mix [52]. This shows that subgrade material treated with 23.5% GGBS can withstand many wetting–drying cycles and still maintain very high CBR values required for use in road construction. The addition of equal proportions of GGBS and BDW exhibited good CBR values usable in road construction at the end of the wetting–drying cycle process. BDW are pozzolanic materials with high alumina/silica content which in the presence of lime can form cementitious gel to increase strength in a mix [53]. Previous studies have also shown that the addition of brick dust increased soil strength by 1.7 to 2.3-fold [51,54]. The lowest CBR value recorded for subgrade 1 composed of 23.5% GGBS was 70% after 10 drying cycles and 52% after 10 wetting cycles. Subgrade 2 composed of 23.5% GGBS recorded a CBR value of 43% after 10 drying cycles and 23% after 10 wetting cycles. Subgrade 1 composed of equal amounts of 11.75% GGBS and 11.75% BDW recorded a CBR value of 58% after 10 drying cycles and 16% after 10 wetting cycles. Subgrade 2 composed of equal amounts of 11.75% GGBS and 11.75% BDW recorded a CBR value of 19% after 10 drying cycles and 15% after 10 wetting cycles. The result shows that CBR values achieved for all subgrades after 10 wetting–drying cycles are very good exceeding minimum 2%, making them suitable for use in road construction. Hence, expansive subgrade materials treated with GGBS and BDW are highly durable and can withstand harsh weather conduction without losing their strength. Figure 4 and Figure 5 show CBR results for subgrade materials after wetting–drying cycles.

### 3.3. The Resilient Modulus

The resilient modulus is considered one of the critical parameters in the design of flexible road pavements as prescribed by some important design guidelines [55,56]. The resilient modulus is often used to estimate pavement layers’ (subgrade, base and sub-base material) behaviour especially when subjected to conditions of cyclic loading. It is also a fundamental mechanical property of materials utilised to explain non-linear stress–strain characteristic of subgrades under the influence of repeated loading [57,58]. According to AASHTO, the resilient modulus is defined as the ratio of the applied cyclic axial stress to the resilient axial strain [59].

**Figure 6:**
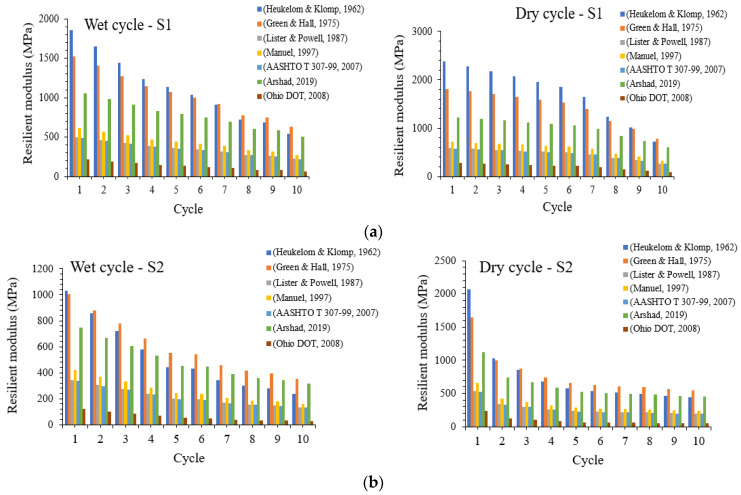
(**a**) Resilient modulus results for subgrade 1 composed of GGBS after 28 days of curing (**b**) Wetting–drying cycle results for subgrade 2 composed of GGBS after 28 days of curing [55,59,60,61,62,63,64].

Several empirical and mathematical relationships have been proposed to estimate the resilient modulus of pavement materials based on the CBR of these materials as tested in the laboratory [55,59,60,61,62,63,64]. In this study, the resilient modulus of natural and stabilised subgrade materials subjected to cycles of wetting and drying derived from previous research as shown in Table 2 is compared as given in Figure 6.

**Figure 7:**
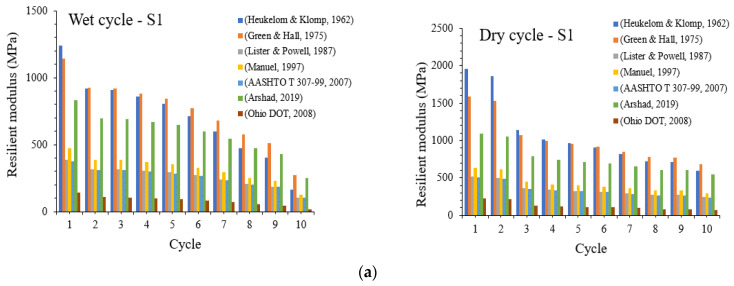

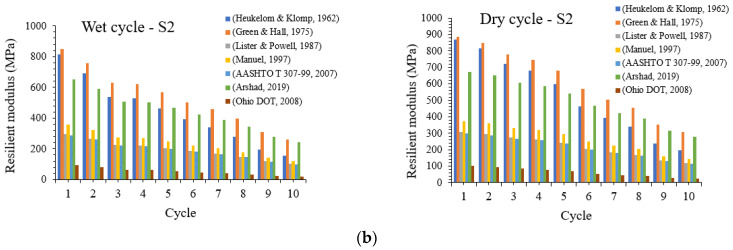
(**a**) Resilient modulus results for subgrade 1 composed of GGBS and BDW after 28 days of curing. (**b**) Wetting–drying cycle results for subgrade 2 composed of GGBS and BDW after 28 days of curing [55,59,60,61,62,63,64].

As indicated in Figure 6, the resilient modulus seems to be generally decreasing with increasing cycles of wetting and drying for stabilised subgrades 1 and 2 without BDW. Across the estimated resilient modulus, values for subgrade 1 are all greater than those for subgrade 2. Moreover, for both wetting and drying cycles, the expression proposed by [62,64] seem to give upper and lower bound values of the resilient modulus, respectively. Meanwhile, the resilient modulus proposed by [61] seem almost indistinguishable. It could also be noticed that the resilient modulus derived from [55,59,60,61,62,63,64] all seem to remain unchanged with the increasing number of wetting and drying cycles especially for stabilised subgrade 1. A similar decreasing trend in the resilient modulus with increasing cycles of wetting and drying is observed for stabilised subgrades 1 and 2 with the inclusion of BDW as shown in Figure 7a,b.

However, some differences are also noticed in the behaviour of the resilient modulus curves when comparing soils stabilised with and without the inclusion of BDW. One of the points of differences is generally noticed with the upper-bound resilient modulus, which is in accordance with the expressions proposed by [61,62] for soils stabilised with BDW included unlike those stabilised without the inclusion of BDW under both wetting and drying cycles. Additionally, for stabilised soil 1 under the drying cycles, the resilient modulus is slightly higher for the first two cycles but does remain unchanged until the 10th cycle. On the other hand, the lower-bound resilient modulus [55,59,64] for stabilised soil 2 with BDW included seems to demonstrate a clear decreasing trend with increasing wetting and drying cycles unlike for stabilised soil 2 without the inclusion of BDW. The results all show the impact of the fluctuating moisture levels from erratic environmental conditions on the performance of stabilised subgrade materials and highlight the improvements gained by blending pozzolanic materials such as BDW and GGBS in enhancing the strength and durability of the stabilised soils. A BDW and GGBS blend is encouraged to increase the durability of the stabilised subgrades.

### 3.4. Microstructural Properties

**Figure 8:**
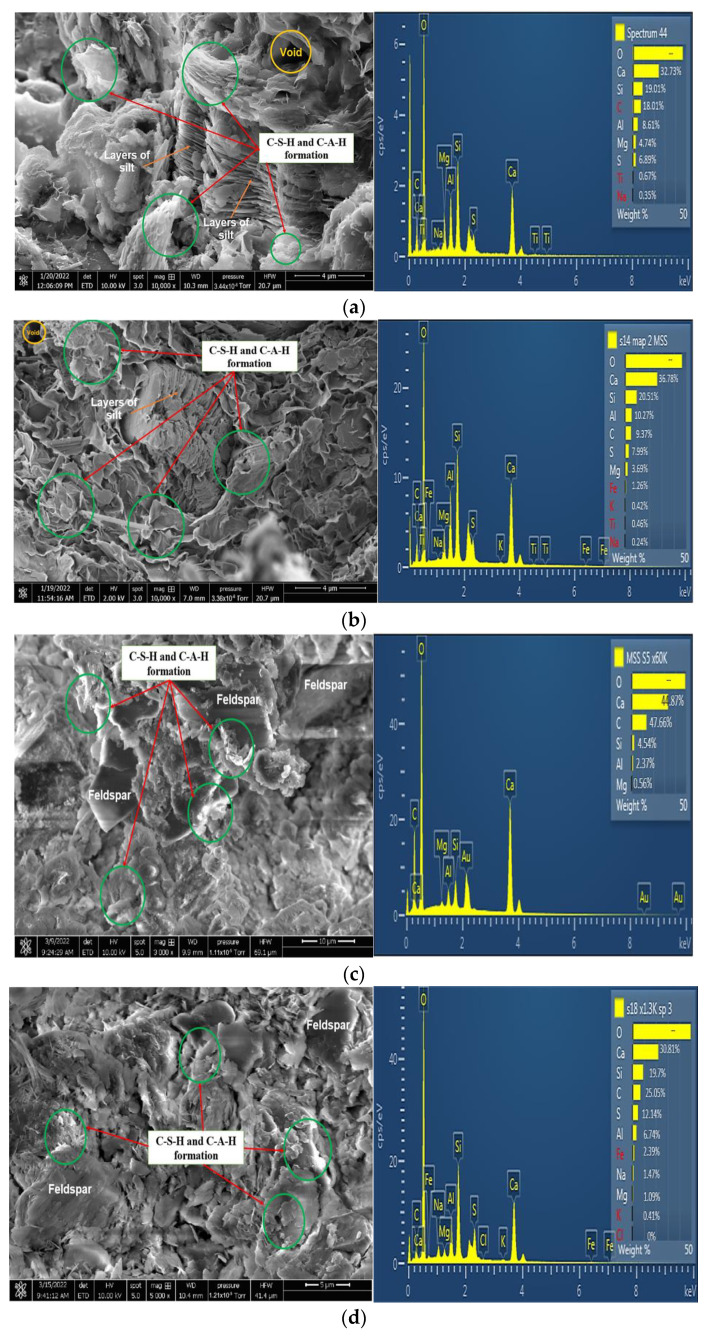
(**a**) SEM and EDX results for subgrade 1 composed of 11.75% GGBS and 11.75% BDW after 28 days curing. (**b**) SEM and EDX results for subgrade 2 composed of 11.75% GGBS and 11.75% BDW after 28 days curing. (**c**) SEM and EDX results for subgrade 1 composed of 23.5% GGBS after 28 days curing. (**d**) SEM and EDX results for subgrade 2 composed of 23.5% GGBS after 28 days curing.

Microstructural analysis conducted showed a high formation of the calcium silicate hydrate (CSH) gel responsible for the increase in strength. Recent studies [53,65] have pointed out that the addition of GGBS and BDW increases the formation of CSH gel due to the rich calcium and pozzolanic characteristic of GGBS and BDW combination. From the result of energy dispersive x-ray analysis, as shown in Figure 8a–d, a calcium (Ca) content of 44.87% was recorded for subgrade 1 treated with the addition of 23.5% GGBS after 28 days of curing. Subgrade 1 composed of 11.75% GGBS and blended with 11.75% BDW recorded 32.73% calcium (Ca) after 28 days of curing. Subgrade 2 composed of 11.75% GGBS and blended with 11.75% BDW recorded 36.78% calcium (Ca) after 28 days of curing. Detailed results of SEM and EDX analysis conducted in this study are shown in Figure 8a–d. From the EDX results, the higher calcium content of subgrades 1 and 2 treated with 23.5% GGBS in combination with lime and cement is logical since GGBS has a higher calcium content, which was further increased with the addition of 2% lime and 2.5% cement. The increase in calcium, however, might not have sustained the increase in strength as calcium alone is insufficient for production of CHS obviously. Reasonable quantities of Si and Al are required to enhance the production of cementitious products. This is seen in the results of the samples with the addition of 11.75% BDW and is an indication of the growth of more cementitious products as shown in Figure 8a,b.

The surface morphology and reduced pores of GGBS- and BDW- treated samples, as shown in the micrographs in Figure 8a,b, align with the CBR results in Section 3.2 which also shows higher strengths for samples treated with GGBS blended with BDW at the end of the 10th cycle. Lower pores are synonymous with increased particle coating ability, which is a function of more CHS flakes as shown in the micrographs of Figure 8c,d. The increased interparticle bonds from a higher pozzolanic activity combined with the initial hydration products from the lime and cement enhanced the durability of the subgrades 1 and 2 when BDW was introduced into the mix. The mineralogical results from EDX also capture a spike in the Al and Si content from 2.37% Al and 4.54% Si to 8.60% Al and 19.01% Si for subgrade 1 when BDW was introduced as shown in Figure 8a,c. Additionally, for subgrade 2, this was from 6.74% Al and 19.70% Si to 10.27% Al and 20.59% Si when BDW was introduced into the binder mix as shown in Figure 8b,d. This strength enhancement is also seen in the lower mass loss recorded from the wetting–drying cycles discussed in Section 3.1. The SEM and EDX results corroborate the fact that the addition of GGBS blended with BDW might be a better combination in dealing with subgrade degradation from cyclic wetting and drying which has become a norm in the light of the rapidly changing environmental condition. The use of GGBS blended with BDW might be considered as an option in the development of resilient road pavement infrastructure to withstand the adverse effects of changing climatic conditions.

## 4. Conclusions

This study focused on the effects of introducing sustainable binders (GGBS blended with BDW) in improving the strength and durability of lime- and cement-treated expansive subgrade for road pavement construction. The bearing capacity of the treated soils has been studied via CBR, under constant wetting and drying cycles. Additionally, microstructural studies have been undertaken to evaluate the changes in the fabric of the stabilised soil at the microlevel. The results of the treated samples containing BDW blends when compared with that of samples without BDW showed strength enhancement and increased durability. Based on the experimental results, the following conclusion can be drawn.

1. Expansive subgrade materials treated with a blend of GGBS and BDW showed higher resistance against degradation at the end of 10 cycles of wetting and drying. The higher strength at the end of the wetting–drying periods indicates that the addition of 11.75% BDW to the binder mix enhanced the resistance of the treated soils against expansion and shrinkage cracks.
2. The strength reduction over the wet–dry cycles was lower for samples treated with a blend of BDW. The addition of BDW increase the production of CHS gels due to the additional pozzolanic activity which increased interparticle bond under the wet–dry cycles.
3. The result of mass loss analysis of the treated soils aligns with the bearing capacity results and the microstructural characteristics indicates that the addition of 11.75% BDW blends into the binder mix is a useful means of lowering the mass loss which occurs through constant swelling and shrinking leading to the breakdown of the bonds. Lower mass loss indicates that subgrade treatment with a blend of BDW has higher resilience and could maintain strength in the event of rapidly fluctuating environmental conditions.

## Figures and Tables

**Table 1:** Chemical Composition of Materials [43].

Oxide (%)	SiO_2_	Al_2_O_3_	Fe_2_O_3_	FeO	MgO	CaO	K_2_O	SO_3_	TiO_2_	Na_2_O	Trace	L.O.I
Bentonite	63.02	21.08	3.25	0.35	2.67	0.65	-	-	-	2.57	0.72	5.64
Kaolinite	48.5	36.0	1.00	-	0.30	0.05	2.15	-	0.06	0.15	-	11.7
Cement	20	6.0	3.0	-	4.21	63	-	2.30	-	-	-	0.80
GGBS	35.35	11.59	0.35	-	8.04	41.99	-	0.23	-	-	-	-
Lime	3.25	0.19	0.16	-	0.45	89.2	0.01	2.05	-	-	-	-
BDW	52	41	0.7	-	0.12	4.32	0.53	0.33	0.65	0.05	-	2.01

**Table 2:** Empirical Relationship for Resilient Modulus Derivation.

SN	Expression	Reference
1	10.33 × CBR	[62]
2	38 × (CBR) ^0.711^	[61]
3	18 × (CBR) ^0.64^	[63]
4	21 × (CBR) ^0.65^	[55]
5	17.6 × (CBR) ^0.64^	[59]
6	49.37 × (CBR) ^0.59^	[60]
7	1.2 × CBR	[64]

## Data Availability

Data can be obtained from corresponding author upon reasonable request.
